# Eligibility for minithoracotomy aortic valve replacement: from Van Praet classification to complex scanner measurements

**DOI:** 10.1038/s41598-022-14994-1

**Published:** 2022-06-29

**Authors:** Yann Barthelemy, Lionel Camilleri, Bruno Pereira, Mehdi Farhat, Lucie Cassagnes, Nicolas d’Ostrevy

**Affiliations:** 1 Department of Cardiac Surgery, Universitary Hospital, Clermont- Ferrand, France; 2 T.G.I., I.P., CNRS, SIGMA, UCA, UMR, 6602 Clermont- Ferrand, France; 3 DRCI, Universitary Hospital, Clermont- Ferrand, France; 4 Department of Medical Imagery, Universitary Hospital, Clermont- Ferrand, France

**Keywords:** Anatomy, Cardiology, Health care

## Abstract

Van Praet proposed a classification to predict the ease of minithoracotomy aortic valve replacement (MT-AVR) based on the position of the aorta in the thorax. We have evaluated the relevance of complex computed tomography (CT) scan measurements to predict the ease of performing a MT-AVR. The first 57 patients who underwent MT-AVR from February 2018 to June 2020 were selected prior to surgery using Van Praet's IA and IB classes. We made additional measurements on aorta position related to the chest and the incision on the preoperative CT scan. The main objective was to correlate complex CT measurements with different operating durations. Van Praet criteria were significantly related to the distance from the center of the aorta to the midline (*p value* < *0.001*), the distance from the center of the aortic ring to the midline (*p value* = *0.013*) and aorto-sternal angle (*p* < 0.001). We did not find a correlation between CT criteria and the different surgical steps durations in patients belonging to Van Praet classes IA and IB. Our cohort of Van Praet class Ia and Ib patients were able to benefit from a MT-AVR without the need for conversion. Complex CT measurements do not provide additional information to predict surgical difficulties. This classification appears to be sufficient to determine a patient's eligibility for MT-AVR, even for a surgeon experienced in sternotomy in his first MT-AVR.

## Introduction

Aortic valve stenosis is the most common valvular pathology in Europe^1^, and its incidence has increased due to the aging of the population. Historically, the replacement of the aortic valve by mechanical or biological prosthesis required a total median sternotomy, allowing a direct view of all cardiac structures^2^. In 1993, the first mini thoracotomy aortic valve replacement (MT-AVR) was performed by Kumar^3^. Since then, numerous studies have shown the advantages of the mini-invasive approach in terms of hospitalization time, aesthetic considerations, and the reduction of postoperative complications^4–7^. Furthermore, automated suture devices (like Cor Knot® (LSI Solutions, Victor, NY) contribute to reducing cross-clamping and cardiopulmonary bypass (CPB) times, even making these times comparable to sternotomy’s standards^8,9^. Glauber's team also demonstrated the ease of performing this procedure, demonstrating the absence of a learning curve, pending an appropriate selection of patients^10^.

**Figure 1:**
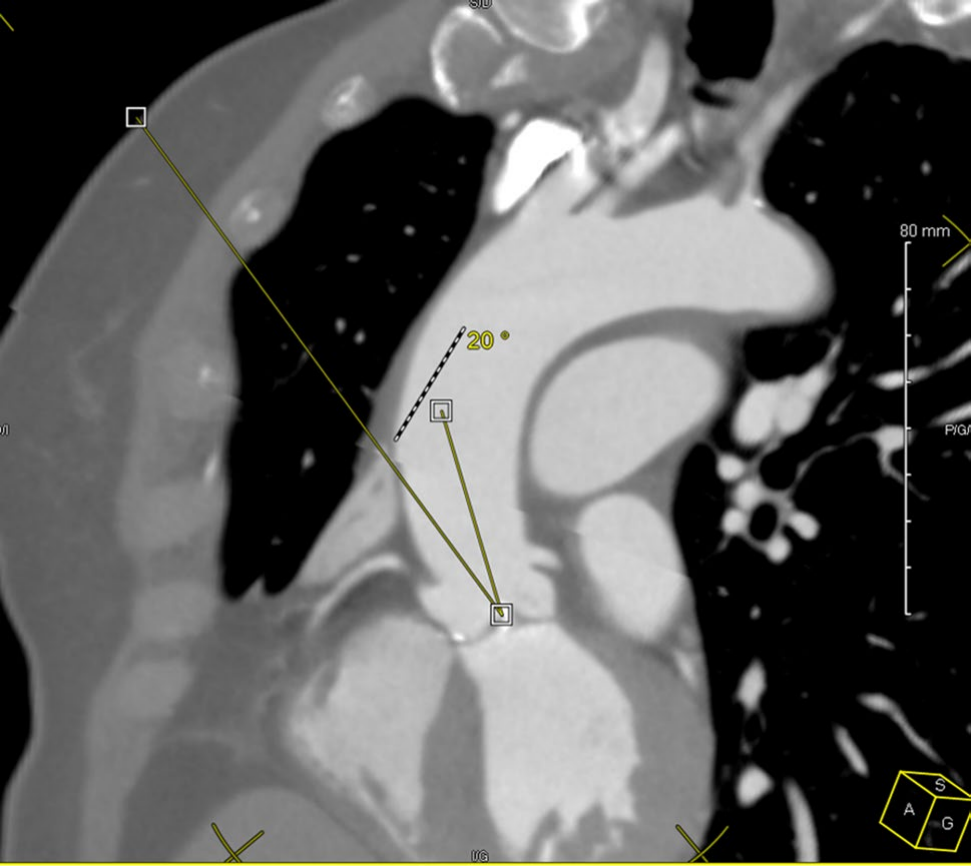
Elattar’s angle measurement.

Several studies^2,11,12^ have attempted to identify criteria predictive of operative difficulty. CT scan with contrast media, which is ideal for anticipating possible difficulties with a single examination, thanks to the different types of visualization and reconstruction it provides, particularly in 3D view and volume rendering^13,14^. Van Praet^11^ proposed a classification based on aorta position in the chest on the CT. Four types of ascending aorta‐sternal relationships at the level of the main pulmonary artery have been defined: Type IA when ascending aorta is completely rightward from the sternum; Type Ib: when ascending aorta is more than 50% located on the right side of the sternum; Type II when more than 50% of the ascending aorta is located underneath the sternum and Type III when ascending aorta is more than 50% located on the left of the sternum. Other studies have evaluated different CT scan measures, such as the angle at the intersection of the line passing through the internal mammary artery (IMA), the center of the aortic ring and the line defined by the center of the aortic ring and the sino-tubular junction (Elattar angle)^12^ (Fig. 1), or through an optimization of intercostal space used, based on the right pulmonary artery position and the right auricle position. In the absence of a demonstrated learning curve, none of these studies, apart from the binary eligibility criteria, were able to describe the ease of performing a valve replacement by minithoracotomy. The multiple operative difficulties, particularly because of the difficulties in exposing the aortic valve, have a potential impact on the different operative times. The aim of our work was to determine whether complex preoperative CT measurements provide additional elements to Van Praet's simple classification for predicting surgical difficulties. We will also validate the criteria proposed by Van Praet on our cohort.

## Materials and methods

We conducted a retrospective monocentric study at the Clermont-Ferrand University Hospital from January 2018 to June 2020, involving a cohort of 57 patients planned for a surgical aortic valve replacement by right thoracotomy. All patients had elective surgery and had undergone preoperative aortic CT scans. All CT scans were obtained on a 64-slice scanner (Discovery 750HD; GE Healthcare®). The acquisitions were performed in supine position, with arms alongside the body. The protocol included a first thoracic acquisition without contrast agent, followed by thoracic aorta angio-tomodensitometry with retrospective gating after injection of 60 ml of Ultravist-370® (Bayer healthcare) at 3.5 ml/s completed with a 40 ml bolus of physiological serum. Bolus detection in the ascending aorta was used for acquisition with a threshold of 160 UH. The acquisition parameters were as follows: 100 kV, intensity modulated between 250 and 550 mA, pitch adapted to the patient's heart rate, cutting thickness 0.625 mm, tube rotation time 0.35 s, detector width 40 mm. The reconstruction was performed by default at 75% of the RR space. The protocol applied in the department considered the eligibility of patients for a minithoracotomy procedure based solely on the Van Praet classification. The criterion for performing minimally invasive surgery was the presence of an ascending aorta that was either located to the right of the right edge of the sternum (corresponding to Van Praet's class IA) or located more than 50% to the right of the sternum (corresponding to Van Praet's class IB)^11^. Patients in Van Praet’s class II or III were considered not suitable for MT-AVR in the initiation of our activity.

The exclusion criteria were:

- The need for surgery other than isolated aortic valve replacement
- The predictable presence of lung adhesions (history of right chest surgery or radiotherapy)
- Extended aortic calcifications^15^
- A distance between skin incision and the aortic ring plane greater than 16 cm^11^
- Dilation of the ascending aorta justifying Bentall’s or ascending aorta replacement
- Compromised femoral and subclavian vascular access, prohibiting the use of extracorporeal circulation cannulation (obliterative arteritis, insufficient diameter, calcifications)

Among our 57 patients who received a MT-AVR, 11 patients were excluded because the preoperative CT scan was not available for reconstruction on our Picture Archiving and Communication System (PACS), and one patient was excluded because he was not classified as being in Van Praet’s class IA or IB.

Study approval: The study was approved, and the written consent of patients waived by the ethics committee of the French Thoracic and Cardiovascular Society (CERC-SFCTCV-2021–07-20-num13_DONI_RAT_AVR). All methods were performed in accordance with the relevant guidelines and regulations.

### Surgical technique

The surgical procedure is similar to the one already described by Bouchot et Van Praet^11,16^. The choice of the intercostal space was made on the preoperative CT scan after confirmation of the Van Praet IA or IB class. The intercostal space of work was the one corresponding to the level of the right pulmonary artery, facing the future site of aortic clamping. In order to limit the size of the thoracotomy, we chose not to perform direct aortic cannulation. However, this approach requires the sacrifice of the right internal thoracic artery which was dissected and ligated. Femoral cannulation was performed through a short horizontal incision at the Scarpa triangle to provide access to the femoral artery and vein. The cannulations were then performed using the Seldinger technique with transesophageal ultrasound to ensure correct positioning of the guidewire, in the superior vena cava for the vein and in the descending thoracic aorta for the artery”.

We used one shot of Custodiol® cardioplegia (EUSA Pharma, Limonest, France) delivered in the ascending aorta. We have chosen not to use a rapid deployment valve in our center. However, to limit the clamping and extracorporeal circulation times, we have systematically used the automatic Cor Knot® (LSI Solutions, Victor, NY) suture device.

### Study times

We measured the following operating times:

- Total operating time (from the first thoracotomy incision to the end of the skin closure)
- CPB time
- Cross-clamping time
- Operative time before cross-clamping (from incision to aortic clamping)

### Follow-up

All immediate post-operative complications and during hospitalization were extracted from the patients' medical records. We also noted late post-operative complications when they occurred in a convalescent home or required rehospitalization.

### Post hoc CT scan measurements

**Figure 2:**
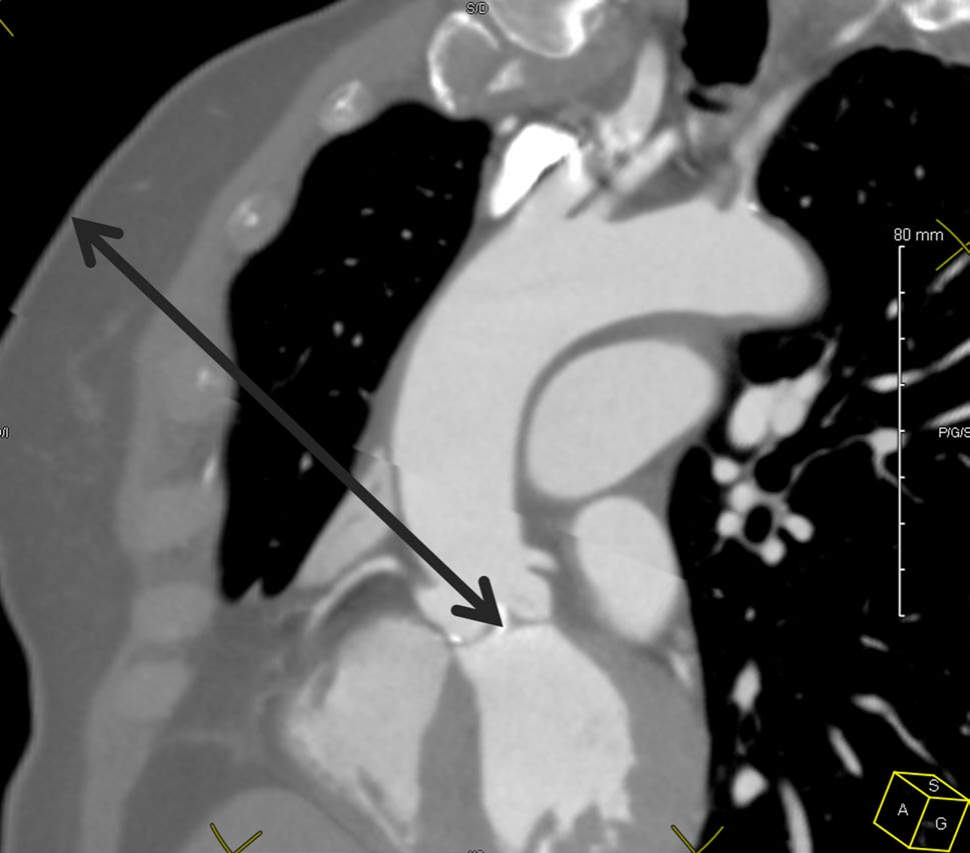
Skin Aortic Ring Center Distance (SARCD) measurement.

All the measurements were performed by a single operator, unaware of the studied times, on a Horizon RadStation® computer (Maincare™). First, we defined the intercostal space of interest corresponding to the right pulmonary artery in axial section (or the overlying space when a costal cartilage was in line with the trunk of this artery). We measured the aorta-skin distances through the IMA, the aorta-sternum distance, the diameter of the aorta, and the angle between a line passing through the right edge of the sternum and the line tangent to the aorta passing through the posterior right angle of the sternum called aorto-sternal angle. We also measured the distance from the center of the aorta to the midline (ACMD), which was recorded positively at the right of the midline and negatively to the left. Then, we measured the distance from the center of the aortic ring to the midline (ARCMD) and the angle passing through the posterior right edge of the sternum and the center of the aortic ring. Finally, on a multiplanar view, we measured the skin-center of the aortic ring distance (SARCD) (Fig. 2). Once this measurement was made, we measured the angle described by Elattar and al.^2^ (Fig. 1), corresponding to the angle formed between the line passing through the center of the ascending aorta and the line passing through internal mammary artery (IMA) and the center of the aortic ring. All the data from the CT measurements are presented in Table 1, according to Van Praet’s IA and IB classes.

**Table 1:** Correlation between Van Praet class and CT scan measurements.

	Van Praet IA Class n = 12	Van Praet IB Class n = 34	*P*-value
Aorta-skin distance (mm)	63 ± 11.2	66 ± 12.9	0.39
Aortic center to median straight-line distance (mm)	23.5 ± 5	9.84 ± 6	**< 0.001**
Aortic diameter (mm)	36.9 ± 7.2	37.1 ± 4.8	0.93
Sternal-aortic angle (°)	167 ± 6	148 ± 11	**< 0.001**
Aorta-sternum distance (mm)	35.1 ± 8.6	27.3 ± 8.9	**0.02**
Aortic ring center to median straight-line distance (mm)	− 5.6 ± 9.2	− 13.5 ± 8.2	**0.01**
Aortic ring center-sternum angle (°)	157 ± 8	154 ± 9	0.35
Skin-aortic ring center Distance (mm)	111.8 ± 10.8	120.8 ± 12	**0.02**
Elattar’s angle (°)	31 ± 5	30 ± 5	0.58

Values in bold are those below the alpha risk threshold set at 0.05.

### Study endpoints

The main objective of this study was to correlate complex CT measurements with different operating times to investigate whether any of them provided additional information to Van Praet's classification to predict operative difficulties. The secondary objective was to further detail Van Praet's classification and to show which precise CT measurements correspond to Van praet's classes Ia and Ib.

### Statistical analysis

Statistical analysis was performed with R software (R Development Core Team, 2010). A two-sided p-value less than 0.05 was considered to indicate statistical significance. The patients were described by frequencies and associated percentages for categorical variables, and by the mean ± standard-deviation for continuous data. The comparisons of continuous variables between groups were performed by the t-test. Categorical parameters were compared between groups using the chi-squared or Fisher’s exact test. Spearman correlation coefficients between CT scan measures and operative times were calculated.

## Results

Among the 57 patients scheduled for aortic valve replacement by minithoracotomy, there was no conversion to sternotomy.

The preoperative characteristics of the patients as well as the operating times are presented in Table 2. Twelve patients were Van Praet class IA patients and 34 were class IB. Age was the only significant difference between the 2 Van Praet’s groups. Regarding the primary endpoints, only the SARCD was statistically significantly related to clamp time (*r* = *0.31, p* = *0.003*) (Table 1). None of the other CT scans measures performed were statistically associated with any of the different durations of the surgical procedures studied.

**Table 2:** Operating data and patient characteristics.

	Van Praet IA Class n = 12	Van Praet IB Class n = 34	*P*-value
Age (years)	70 ± 4.8	66 +/− 9.8	0.05
Weight (kg)	79.7 ± 13.5	78.5 ± 11.98	0.78
Height (cm)	171 ± 7.4	171 ± 7.1	0.93
Body Mass Index (kg/m^2^)	27 ± 2.5	27 ± 3.5	0.72
Euroscore2 (%)	1.03 ± 0.004	1.01 ± 0.004	0.88
Preoperative creatinine, (µmol/L)	83 ± 11	85 ± 15	0.66
LVEF, preoperative (%)	60 ± 8	63 ± 10	0.23
ICU stay (hours)	71 ± 52	55 ± 40	0.35
Hospitalization stay (hours)	175 ± 143	137 ± 85	0.41
Total operating time (min)	217 ± 37	214 ± 36	0.80
CPB time (min)	125 ± 19	129 ± 23	0.55
Cross-clamping time (min)	78 ± 10	85 ± 14	0.06
Intervention time before cross-clamping (min)	73 ± 17	72 ± 14	0.86

*CPB* cardiopulmonary bypass, *ICU* intensive care unit, *LVEF* left ventricle ejection fraction.

Regarding the secondary endpoint of our study, the Van Praet criteria were significantly correlated with center of the aorta to the midline (ACMD: *p value* < *0.001*), aorto-sternal angle (*p value* < *0.001*), aorta-sternum distance (p = 0.015) center of the aortic ring to the midline (ARCMD: *p value* = *0.013*) and skin-center of the aortic ring distance (SARCD: p value = 0.02) (Table 3).

**Table 3:** Correlation between CT scan measurements and intervention times.

n = 46		CPB Time	Cross-clamping time	Operative time before cross-clamping	Total operative time
Aorta-skin distance	*p-value*	0.0808	0.082	0.796	0.572
Aortic center to median straight line distance	*p-value*	0.0675	0.0891	0.966	0.513
Aortic diameter	*p-value*	0.722	0.984	0.23	0.695
Sternal-aortic angle	*p-value*	0.892	0.538	0.618	0.812
Aorta-sternum distance	*p-value*	0.478	0.885	0.393	0.997
Aortic ring center to median straight line distance	*p-value*	0.224	0.42	0.676	0.836
Aortic ring center-sternum angle	*p-value*	0.793	0.602	0.408	0.744
Skin-aortic ring center distance (SARCD)	*p-value*	0.0743	**0.0331**	0.814	0.261
	*r*		**0.31**		
Elattar’s angle	*p-value*	0.775	0.987	0.869	0.377

Values in bold are those below the alpha risk threshold set at 0.05.

Postoperative complications are described in Table 4.

**Table 4:** postoperative events and complications.

Postoperative event	n (%)
Transient ischemic attack and neurologic event	2 (4%)
Heart rhythm trouble	4 (8%)
Pneumothorax	1 (2%)
Surgical site infection and other infections	2 (4%)
Surgical site infection	0 (0%)
Hemothorax	1 (2%)
Blood transfusion	3 (6%)

## Discussion

**Figure 3:**
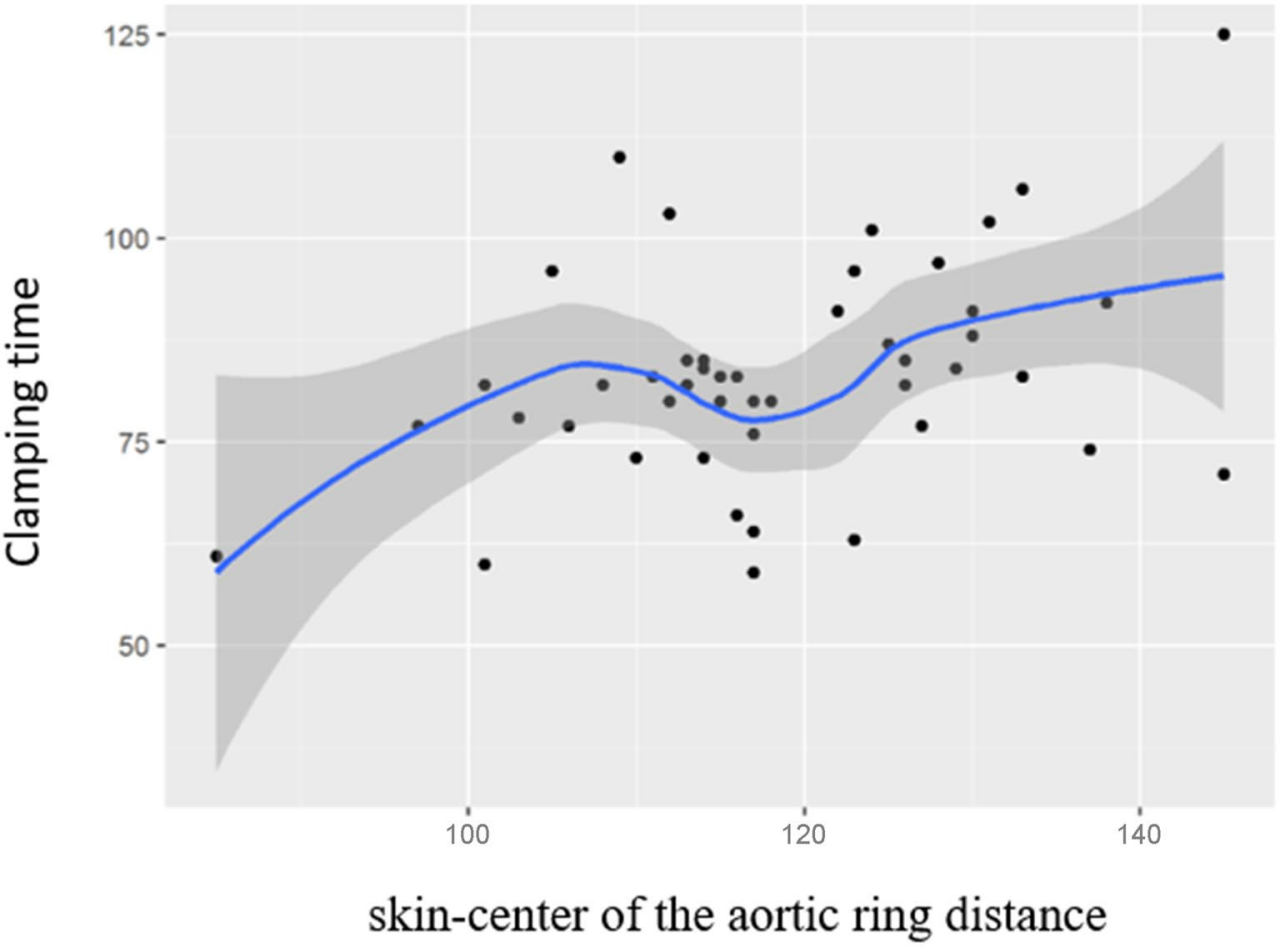
Correlation between clamping time and skin-center of the aortic ring distance.

In our study, we found a significant correlation between skin-center of the aortic ring distance and cross-clamping time, but the clinical relevance of this correlation seems to be insufficient^17^ (Fig. 3), probably due to our rigorous pre-selection of patients as indicated for MT-AVR with a skin-to-aortic ring distance under 16 cm as Van Praet suggested in his description of their selection criteria^11^. We also found that aortic measurements from the CT scan (center of the aorta to the midline, center of the aortic ring to the midline, aorta-sternal angle) were correlated with the Van Praet classification. This statistical correlation likely stems from the fact that these measures define the position of the ascending aorta in the thorax. Surprisingly, we found a significant difference among Van Praet groups with respect to age. This observation may be related to aortic morphologic changes already described in the literature^18,19^.

Int their publication, the angle of Elattar seems an important factor in predicting operative difficulties, particularly of the time required for CPB and cross-clamping^12^. However, our results did not reveal a similar correlation, probably because our study only included patients that were classified into Van Praet classes IA and IB. In his work, Elattar proposed that Elattar angles greater than 38° predict surgical difficulties, but only two of our patients met this criterion. This is likely because Van Praet's criteria selects patients with Elattar angles lower than 38°. Probably the Elattar angle has a threshold effect and below this threshold, there is no correlation between the value of the angle and the operating difficulties.

Extracorporeal circulation and clamping times less than 240 min and 150 min, respectively are protective factors against morbidity and mortality, regardless of the procedure performed in the litterature^20^. However, there is no consensus on these cut-off values, considering that the risks of morbidity and mortality can appear as soon as after 60 min of clamping^21^.

The use of sutureless or rapid deployment valves was proposed in an effort to limit surgical durations. Because of their additional cost, but also in order to use the valves best proven durability, we chose to use stented classic surgical valves. The use of the Cor-Knot device limits the increase of the cross clamping time.

In our cohort of patients pre-screened according to Van Praet's criteria, the total time for median cross clamping and CPB was well below the proposed limits^20^. Although their work focuses on rapidly deploying valves, Murzi demonstrated that the use of a minithoracotomy approach was not associated with an identifiable learning curve for a surgeon experienced in aortic valve replacement by sternotomy^10^. This element is debated in the literature. Several studies report a learning curve in minithoracotomy aortic replacement. However, they do not detail the type of valve or the sternotomy experience of the surgeon at the initiation of the minithoracotomy surgery program^7,22^.

Postoperative complications were relatively rare, with no cases of stroke despite systematic femoral cannulation.

This association has, however, been widely described in the literature^23^. The careful selection of patients we considered eligible for this technique most likely contributed to these good results. Our study, however, had limitations; we conducted a monocentric study on a small cohort with CT scans performed in forced inspiration (scanner protocol) and not at the end of expiration (which would correspond to the patient under CPB).

## Conclusion

Finally, complex measurements on the pre-operative CT scan only seem to provide an unnecessary analysis according to Van Praet's criteria. Belonging to Van Praet's classes Ia and Ib is a robust and predictive criterion of relative ease of realization of MT-AVR including by a surgeon during the learning curve. This single selection criterion is also sufficient to predict operating, CPB and cross clamping times below the literature thresholds. Therefore, minimally invasive aortic surgery can be performed with acceptable risks for the patient after a good selection of patients corresponding to Van Praet's classes IA and IB.
